# The neural crest cell hypothesis: no unified explanation for domestication

**DOI:** 10.1093/genetics/iyab097

**Published:** 2021-07-10

**Authors:** Martin Johnsson, Rie Henriksen, Dominic Wright

**Affiliations:** 1 Department of Animal Breeding and Genetics, Swedish University of Agricultural Sciences, Uppsala 750 07, Sweden; 2 IFM Biology, Linköping University, Linköping 58183, Sweden

## Introduction

A few years ago, Wilkins *et al.* (2014) advanced the neural crest cell hypothesis of domestication as an explanation for the “domestication syndrome” in animals, which refers to a collection of characteristic traits associated with domestication. Since then, the neural crest cell hypothesis has risen to become arguably the most popular explanation for the genetics of the initial phase of the domestication process (Zeder 2017; Thomas and Kirby 2018; Kikusui *et al.* 2019; Zanella *et al.* 2019). 

Neural crest cells contribute to many different parts of the vertebrate body. They are the origin of most of the peripheral nervous system including parasympathetic and sympathetic ganglia, the enteric nervous system and sensory ganglia; sensory receptor cells in the skin; pigment cells, except in the retina; endocrine cells in the adrenal medulla and thyroid; the frontal part of the head, including bone, connective tissues, and muscle; and parts of the cardiovascular system (reviewed by Dupin *et al.* 2006). The neural crest cell hypothesis was motivated by a search for common factors between tameness and other domestication syndrome traits, and an apparent similarity between domestic animals and humans with neurocristopathies, posited by Wilkins *et al.* (2014). They hypothesized that if tameness is mainly caused by changes in the adrenal glands, selection on tameness might change neural crest development to cause correlated changes such as brain size reduction, floppy ears, altered facial shape, and altered pigmentation.

The hypothesis entails that a reduction in neural crest cell proliferation and migration is a core genetic mechanism of early domestication. This implies that the genetic architecture of domestication is by necessity pleiotropic, with variants in genes affecting neural crest development and distribution causing multiple domestication traits. The authors also suggest that neural crest cell function is polygenic, affected by many genetic variants with individually small effects. Furthermore, they have suggested that it involves epigenetic inheritance, genetic assimilation, or epistatic interaction between genes where changes to the neural crest may potentiate later evolution of other domestication traits (Wilkins 2020). 

Because pleiotropy is at the core of the neural crest cell hypothesis of domestication, we need to clarify what we mean by this term. Paaby and Rockman (2013) distinguish between when *genes* have multiple functions, for example in different pathways, reactions or protein–protein interactions, and when *alleles* have effects on multiple traits, for example, by causing a heritable syndrome. As this distinction pertains to the neural crest cell hypothesis, many genes involved in the neural crest are pleiotropic in the sense that they participate in multiple developmental processes. However, for the neural crest cell hypothesis to work, there also have to be pleiotropic alleles, selected during domestication, that affect multiple domestication traits through their effect on the neural crest.

The development of neural crest cells is regulated by a complex gene regulatory network that spans multiple cell types and temporal phases (reviewed by Betancur *et al.* 2010; Rogers *et al.* 2012). Neural crest cells originate at the border of the neural plate. After induction, they undergo an epithelial-to-mesenchymal transition (where they lose their epithelial properties), delaminate (split off) from the neural tube, and migrate to their target locations where they differentiate. Neural crest induction happens in response to secreted signaling molecules [including Bone morphogenetic proteins (*BMPs*), Fibroblast growth factors (*FGFs*), and *Wnt* ligands] secreted by surrounding tissues, and Notch signaling, which is based on cell–cell contact. These signals lead to the expression of neural plate border specifiers (homeobox genes including *Msx1* and *Pax3* and zinc finger *Zic* genes), a group of transcription factors that trigger neural crest specifiers. Neural crest specifiers are the key transcription factors expressed in neural crest cells themselves before and during migration (including *Snai1/2*, *Id*, *cMyc*, *Sox9*, and *Sox10*). Furthermore, there is a set of genes (including *FoxD3*, *Snai1/2*, and *Sox5*) that regulate how neural crest cells delaminate and go through epithelial-to-mesenchymal transition, needed for them to migrate. This process involves changes to the extracellular matrix, and regulation of cell adhesion molecules including cadherins and integrins. When arriving at their destination, neural crest cells typically stop expressing neural crest specifiers, but some of them keep being expressed and serve to regulate differentiation (*e.g.*, *Sox10* in the peripheral nervous system and in melanocytes). Pleiotropic genetic variants acting on neural crest cell development, which is what the neural crest cell hypothesis predicts, could affect any of these processes, up until but likely not including the regulation of differentiation at the target site. Since the publication of Wilkins *et al.* (2014), several selective sweep scans in domestic animals have reported enrichments of neural crest-related genes and individual candidate genes related to the neural crest (reviewed by Wilkins 2017).

There are several weaknesses in the evidence for the neural crest cell hypothesis that, in our view, make it implausible and premature as an explanation for domestication traits and as a unifying hypothesis for the domestication syndrome. In this letter, we present three arguments against the neural crest cell hypothesis as a unified explanation for traits under domestication. These are:

1. Several of the key traits potentially explained by neural crest cell mechanisms are not parts of a universal domestication syndrome.
2. To the extent that there is a domestication syndrome, that does not imply a universal genetic mechanism.
3. Even if we postulate a universal genetic mechanism, the evidence that this mechanism is neural crest cell proliferation and migration is indirect and weak.

We then examine in detail some of the evidence from genome scans that is purported to support the neural crest cell hypothesis, which is weaker than it first might seem. Finally, we suggest some future research directions for the genomics of domestication in the light of these arguments.

## Several of the key traits potentially explained by neural crest cell mechanisms are not parts of a universal domestication syndrome

The idea of a domestication syndrome is some 40 years older than the neural crest cell hypothesis, and not dependent on any particular genetic mechanism. However, for the neural crest cell hypothesis to have anything to explain, there must be an at least somewhat universal domestication syndrome. The term “domestication syndrome” was popularized for animals by Price (1984, 2002), though the original idea came from plant biology (Faegri 1981; attributed by Faegri to Swiss botanist Albert Thellung). At its core, the domestication syndrome consists of alterations in behavior (increased tameness), body size and composition, brain size and composition, and color [though even this was not considered initially (Price 2002; Wright *et al.* 2020)].

These core domestication traits have been consistently altered in almost every domestic animal, but the type and direction of changes are not always consistent in all domestic animals. If we take brain size and composition as an example, the telencephalon is most consistently increased in relative size to the rest of the brain in pigs, llama, and alpaca (Kruska and Röhrs 1974; Kruska 1982, 1988), but not in the case of sheep (Ebinger 1974). In the dog, brain composition is highly variable between breeds (Hecht *et al.* 2019). Similarly, in domestic birds the cerebellum and cerebral hemisphere have consistently increased relative to the rest of the brain in domestic chickens, geese, pigeons, and turkeys, but not in domestic ducks (Ebinger and Löhmer 1984, 1987 ; Ebinger 1995; Ebinger and Röhrs 1995; Gille and Salomon 2000; Rehkämper *et al.* 2008; Henriksen *et al.* 2016). There is a wide degree of variation in the extent of allometric brain size reduction between domestic species, with pigeons only showing a 7% reduction, whereas turkeys exhibit a 35% reduction. Body composition and skull changes also show a similar pattern. Sánchez-Villagra *et al.* (2017) looked in detail on skull morphology in pairs of wild and domestic animals and Wilson (2018) at developmental trajectories of skull morphology within domestic species. Neither found a single universal pattern. That is, skull morphology has changed in many domestic animals, but it has not changed in the same way.

The domestication syndrome is often expanded to include a multitude of other traits, including hair and feather morphology, dentition, and many others characteristics (Sánchez-Villagra *et al.* 2016; Lord *et al.* 2020; Wright *et al.* 2020). Several of the key traits that inspired the neural crest cell hypothesis—facial morphology, floppy ears, tail shape, and so on—should not be considered part of the domestication syndrome proper, because there is no good evidence that they are universal in domestic animals. We also need to keep in mind that differences observed between breeds of the same species may have happened during breed formation and selective breeding, and are not necessarily reflective of changes during the initial phase of domestication. For example, the floppy ears of certain dog breeds and experimentally domesticated foxes may or may not be due to changes in the regulation of neural crest cells; this is currently not known. However, even if they were, the occurrence of floppy ears is not a universal feature of domestic animals. The same goes for most other proposed domestication traits, as reviewed in detail by Sánchez-Villagra *et al.* (2016) and Lord *et al.* (2020).

While there are knowledgeable scientists who argue otherwise (Lord *et al.* 2020), we believe that the domestication syndrome is a meaningful observation that warrants explanation, when restricted to core traits that are universal. One can certainly imagine neural crest-driven mechanisms for these core features: tameness might be caused by endocrine effects in the adrenal glands; brain size and composition might be indirectly affected by the size and shape of the cranium. However, the explanatory power of the neural crest cell hypothesis shrinks when we keep in mind that many of the features it aims to explain are not universal features of domestication, but specific to the history and biology of particular species.

## To the extent that there is a domestication syndrome, that does not imply a universal genetic mechanism

Even where traits are consistently altered in domestic species, or change together in experimental domestication studies, the mechanism may not be pleiotropic alleles. In support of the neural crest cell hypothesis, Wilkins (2020) argues that “The simplest way to view the situation is that a GRN [gene regulatory network], affecting all these traits, is involved and that mutations in relatively upstream elements in this GRN account for many of the traits ….” However, given recent developments in quantitative genetics, explanations based on a single gene regulatory network appear to be the exception rather than the rule. Recent evidence from quantitative genetics (Boyle *et al.* 2017) in humans suggests that polygenic traits tend to be omnigenic. That is, heritable variation in traits is not mainly due to a core biological process or pathway, but causative variants may act on almost any gene expressed in a relevant cell type, mediated by trans-regulatory variation (Liu *et al.* 2019).

Experimental domestication studies provide a way to isolate the effect of selection on behavior, and reveal genuine correlated responses. However, a correlated response does not imply pleiotropy. The most famous domestication experiment is the farm fox experiment of Belyaev and co-workers (reviewed by Belyaev 1979; Trut *et al.* 2009; Lord *et al.* 2020). The experiment has been widely claimed (for example by Wilkins *et al.* 2014) to support a universal domestication syndrome caused by selection on behavior, and it is one of the main pieces of evidence that strong pleiotropy underpins domestication. However, there are several issues that have been raised that cast doubt as to how applicable this experiment is to a domestication model, and also how the traits themselves are correlated and documented. Importantly, the farmed fox experiment was actually founded on already domesticated animals (Statham *et al.* 2011; Lord *et al.* 2020). The co-occurrence of domestication syndrome traits in natural domesticates may be due to selection on multiple traits, and recent evidence suggests that this might be the case even for the farm fox domestication experiment. As such, the different domestication features that were present in the selected lines have already been present in the population and not developed as a result of selection.

Linked selection can cause apparent pleiotropy between unrelated genes, when causative variants for different traits are located close to each other in the genome, even if the variants themselves share no functional similarity. This mechanism can potentially also cause parallel changes between different species, to the extent that gene synteny is preserved between species. Recent analyses of experimental evolution data from *Drosophila* suggest that linked selection is a substantial driver of allele frequency change in selection experiments (Buffalo and Coop 2020). This effect is likely to be even more pronounced in studies with smaller effective population sizes, and thus, interpreting correlated selection in the farm fox experiment as evidence of pleiotropy is problematic.

Furthermore, there are other plausible explanations for correlations between domestication traits than a shared origin in the neural crest. For example, selection on fearful behavior (one certain universal feature of domestication), may affect seasonality through changes in the central nervous system, upstream in the hypothalamus-pituitary-gonad axis. A downstream effect of selection for tameness on the endocrine system was initially Belyavev’s own preferred hypothesis (Belyaev 1979), before it was re-interpreted as a downstream effect of changes to the neural crest by Wilkins *et al.* (2014).

Direct evidence of pleiotropic alleles underlying the domestication syndrome is sparse. Genetic mapping studies of domestic species can inform about the extent of pleiotropy and close linkage between different domestication traits, albeit with the limited genomic resolution and power to detect small effects inherent in linkage mapping studies. Evidence from chickens shows that domestication traits initially colocalize with one another when an F_2_ inter-cross population is considered, but when further generations are added and hence more recombinations between loci accrue, these loci are found to actually be distinct and linked rather than pleiotropic (Johnsson *et al.* 2014). Similarly, linkage mapping of rat lines selected for high and low aggression, initially by Belyaev himself, (Albert *et al.* 2009) found only weak linkage between domestication traits. Genetic mapping of tameness in the farm foxes themselves do not address the question, but did detect one locus associated with different aspects of tameness behavior (Kukekova *et al.* 2011). Future genetic dissection of this association might detect, or rule out, pleiotropic effects on other traits. When expression quantitative trait loci are considered (in the chicken and rat models) a lack of gene-regulatory hotspots are once again observed, again providing no evidence of pleiotropy (Heyne *et al.* 2014; Johnsson *et al.* 2016; Höglund *et al.* 2020).

In summary, even if we assume that there is a core domestication syndrome responding to selection on behavior, as the farm fox experiment suggests, that response may be due to linked selection, or by pleiotropy that is mediated through locus and trait-specific mechanisms that may or may not involve the neural crest. The neural crest cell hypothesis may be a simpler explanation than scenarios potentially involving many biological processes, but based on both quantitative genetics and molecular biology, we have little reason to expect complex traits to be underpinned by a single biological process.

## Even if we postulate a universal genetic mechanism, the evidence that this mechanism is neural crest cell proliferation and migration is indirect and weak

Recent genomic studies (Carneiro *et al.* 2014; Pendleton *et al.* 2018; Wang *et al.* 2018) have found evidence of selection at a few handfuls of genes that have molecular functions associated with the neural crest, but that in itself is not good evidence that neural crest cell proliferation and migration are underlying the domestication syndrome. For that, we would need evidence that these alleles were selected specifically for their function in the neural crest and that they cause pleiotropic effects on other traits. There are three main reasons why the available selection mapping data provide only weak evidence for the neural crest cell hypothesis.

First, there are limitations to selection mapping that provide an inherently biased view of adaptation: Selective sweep scans are limited in their ability to detect polygenic adaptation of complex traits that may be due to small shifts in allele frequency at many loci (Pritchard and Di Rienzo 2010). Selective sweep scans also struggle with adaptation from standing variation (Hermisson and Pennings 2005), which should be expected to contribute to domestication both in natural domesticates and in experimental population where there is little time for new mutations to occur. Thus, selective sweeps likely represent simpler traits or major loci for complex traits. This is consistent with selective sweep signals at known genes responsible for monogenic or oligogenic traits in domestic animals, *e.g.*, the *BCO2* skin pigmentation variant in chickens (Rubin *et al.* 2010), pigmentation genes in cattle (Qanbari *et al.* 2014), and the *NR6A1* gene associated with vertebrae number in pigs (Rubin *et al.* 2012). Selective sweeps represent recent adaptation, meaning that sweeps in natural domesticated animals are likely to reflect more recent events such as breed formation and improvement. For example, an ancient DNA study shows how two strong sweeps in the domestic chicken, at *TSHR* and *BCO2*, both were selected much later than early domestication (Flink *et al.* 2014; Loog *et al.* 2017). It stands to reason that the same may be true for other “domestication sweeps,” even with genuine signals of selection.

Second, when we observe a number of genes with a certain function in selective sweep data, how noteworthy is this? We have no appropriate statistical model for how many neural-crest related genes will be observed in a genome scan. Annotation term enrichment tests are known for their false-positive rates and researcher degrees of freedom when applied in a genome scan context (Pavlidis *et al.* 2012), allowing significant gene set enrichments and biologically plausible stories to be spun out of noise. Therefore, gene set enrichments from selective sweep scans should be viewed with suspicion. Furthermore, the interpretation of pathway enrichments is problematic because developmental genes are highly pleiotropic. The same genes and pathways are re-used in many developmental contexts. For example, the same signaling pathways (*BMP*, *FGF*, and *Wnt*) have different functions during different phases of neural crest development (Taneyhill and Bronner-Fraser 2005); temporal context matters. The same transcription factor (*Sox2*) maintains neural progenitor identity in the developing nervous system, inhibits neural crest cell formation, and maintains pluripotency in embryonic stem cells (Avilion *et al.* 2003; Mandalos and Remboutsika 2017); cellular context matters. Therefore, multiple underlying processes can give rise to the same enriched pathways, and limit our ability to infer process from pattern. Furthermore, even gene expression itself is not an indicator of cell type or cell lineage, especially considering the extreme pleiotropy of many of these transcription factors. Just because some of the factors that act as neural crest specifiers may be expressed in a particular tissue, this is certainly no guarantee that the cells involved are either neural crest cells or derived from the neural crest.

Third, there is no direct functional evidence for the core prediction of the neural crest cell hypothesis, namely that these selected alleles have pleiotropic effects on domestication traits. Selective sweep scans cannot provide any such evidence in principle, as they do not use any trait information. When genome scans reveal selection at genes that have multiple molecular functions, that is not necessarily good evidence that the selected variant had pleiotropic effects on multiple traits. A selective sweep in or close to a key developmental regulatory gene might affect its expression in any of many different cell types, which need not be functionally connected. We will elaborate on this point in the next section, which deals with some of the candidate domestication genes potentially related to the neural crest.

In summary, enrichment of neural crest-related genes in selective sweeps are not good evidence for the neural crest cell hypothesis of domestication. This is because selection mapping is unsuited to study complex traits in early domestication, because enrichment tests are unsuited to interpreting selective sweep scans and developmental processes, and because evidence of pleiotropic alleles, as implied by the neural crest cell hypothesis, is lacking.

## Examples of the evidence from genome scans

We will examine in detail the evidence for the neural crest cell hypothesis found in two studies of naturally domesticated animals [rabbits (Carneiro *et al.* 2014) and dogs (Pendleton *et al.* 2018)], and then in a selection study of the farm fox experiment (Wang *et al.* 2018). In the case of Carneiro *et al.* (2014) the authors themselves did not discuss the neural crest cell hypothesis; this reinterpretation is due to Wilkins (2017). One should keep in mind that genetic and selection mapping are challenging and have limited power, and the absence of evidence is not evidence of absence. We are not criticizing the population genetic methods in these studies, or arguing that these data are strong evidence in favor of any other mechanism; what we argue is that they provide only weak evidence for the neural crest cell hypothesis.

### *SOX2* and *PAX2* in rabbits

*SOX2* and *PAX2* are two transcription factors with developmental roles associated with selection signals in the domestic rabbit (Carneiro *et al.* 2014). Wilkins (2017) interpreted them as support for the neural crest cell hypothesis; in the original publication, they were discussed in terms of neural differentiation. Both genes are involved in the neural crest gene regulatory network (Williams *et al.* 2019), but this is by no means their sole function. *SOX2* is a pluripotency factor that maintains embryonic stem cell identity (Avilion *et al.* 2003). With regard to the neural crest, *SOX2* is expressed in the neural tube before neural crest induction and inhibits neural crest formation (reviewed by Mandalos and Remboutsika 2017). Expression is reduced in neural crest cells after induction, and suppressed during migration, but again upregulated in a subset of neural crest cells that are going to become peripheral neurons or glia cells (Wakamatsu *et al.* 2004). Outside of the neural crest, *SOX2* maintains neural progenitor status by preventing differentiation into neurons. It is expressed in multipotent neural progenitors of the central nervous system, where it inhibits differentiation (Graham *et al.* 2003). *PAX2* is expressed during development of the central nervous system, including in the ventricular zone (a transient embryonic layer containing neural stem cells) and the developing eye and ear (Nornes *et al.* 1990). Expression analysis in zebrafish (Kelly and Moon 1995) and double knockouts in mice (in conjunction with *Pax5*) suggest that *PAX2* contributes to patterning of the central nervous system (Urbánek *et al.* 1997). However, a recent study of the neural crest gene regulatory network by RNA and chromatin sequencing found evidence that *PAX2* is involved in regulating neural crest cell development, despite previously being associated mostly with neurons (Williams *et al.* 2019).

### *FGF and Wnt signaling* in dogs

A selective sweep scan in dogs (Pendleton *et al.* 2018) highlighted selection on *FGF* and *Wnt* signaling pathways as potential support for the neural crest cell hypothesis. The evidence consists of three enriched *Wnt*-signalling related terms (*noncanonical Wnt signaling pathway; Wnt signaling pathway, planar cell polarity pathway;* and *regulation of noncanonical Wnt signaling pathway*), out of hundreds of enriched terms, and a handful of other genes (*TCF4*, *RRM2*, *NPHP3*, and *LGR5*) out of hundreds of genes. *Wnt* signaling is a central developmental pathway. Among many other processes, a *Wnt* gradient is involved in anterior–posterior patterning of the neural tube, regionalization and patterning of the brain, and wiring of the central nervous system (reviewed by Ciani and Salinas 2005). *FGF* signaling, on the other hand, was not enriched, but supported by the presence of *FGF13*, *FGF18*, and *FGF1* in selected regions. *FGFs* are involved in almost all tissues, throughout both development and adult life (Ornitz and Itoh 2015).

### *Wnt* and *protocadherin* in farm foxes

The farm fox selection study of Wang *et al.* (2018) cites support for the neural crest cell hypothesis in its significance statement and in its abstract, but getting to the main text, the support amounts to the presence of *Wnt3*, *Wnt4*, and *protocadherin* among genes with significant allele frequency change. Again, *Wnt* signaling contributes to the development of near every part of the body (Wodarz and Nusse 1998). Protocadherins play a role in neural crest cells, but they are also involved in central nervous system development, neural identity, and connectivity (Sano *et al.* 1993; Frank and Kemler 2002).

In summary, calling the genes that have been highlighted here “neural crest cell genes” is true but incomplete. They are involved in a multitude of developmental processes in a wide range of cell types, and any evidence that their role in domestication is mediated by the neural crest is absent. This type of reasoning would be like describing anything French as “Paris-associated” because Paris is the capital of France. It is not wrong, just uninformative. Several of the candidate genes and pathways have roles both in the development of the central nervous system and of the neural crest. Thus, selection on the central nervous system and on the neural crest cell might give rise to similar pathway enrichments.

Each of the papers discussed above is sufficiently cautious with its interpretation, making it clear to anyone reading the full paper that the evidence consists of a few genes or a few enriched Gene Ontology terms. At the same time, Pendleton *et al.* (2018) and Wang *et al.* (2018) especially highlight the neural crest cell hypothesis, possibly because it is the one “unifying hypothesis” about domestication that is popular at the moment. The unfortunate outcome is that the nonspecialist reader comes away with the impression that the neural crest cell hypothesis of domestication is well-supported by evidence from multiple species, when the evidence is at best ambiguous and indirect.

## Future directions for research

We hope that a reconsideration of the neural crest cell hypothesis will lead to more fruitful research than continuing to mine genomic datasets for evidence of neural crest cell genes. While there may be many different fruitful avenues of research and possible alternative hypotheses about correlated traits under domestication, we suggest some possible directions for genomics of domestication:

- *Comparative population genetics*: The amount of data and the sophistication of population genetic methods for detecting selection have grown steadily since the selection mapping studies of domestication. New methods are better at detecting sweeps from standing variation (Stephan 2019), and at dealing with complex demography. Thus, it is possible to integrate the population genetic data that is already out there with uniform processing, modern population genetic methods, and modeling of demography. That would make it possible to compare sweeps between species and see which genes or pathways are universal. For example, there is evidence of shared signatures of selection during domestication between sheep and goats (Alberto *et al.* 2018). While the fundamental limitations of selection mapping still apply, this is bound to be more accurate than comparing gene lists from studies that generated them in different ways.
- *Functional genomics of domestication*: The development of functional genomic technologies has the potential to be a boon for domestication research. It is now possible to build atlases of gene expression and chromatin in time courses of embryos (*e.g.*, Villar *et al.* 2015; Cardoso-Moreira *et al.* 2019) and at cell-type resolution with single-cell technologies. If applied to wild and domestic animals, such studies could tell us what transcription factors, enhancers, and promoters differ between wild and domestic animals. If applied to the neural crest cells, for example (as is done by Williams *et al.* 2019) in a comparative settings, we would learn if the timing and differentiation of neural crest cells are meaningfully different between wild and domestic animals.
- An upside of the neural crest cell hypothesis of domestication is that it has increased the interest in development among domestication researchers. There have been proposals to estimate differences in the neural crest function in domestication directly for example in chickens (Wilkins *et al.* 2014). Since these studies have not yet materialized, we suspect that performing this kind of functional studies is easier said than done. There is also a difference in approach: Developmental genetics chiefly works bottom-up from a known set of important genes and pathways, and towards systems. Selection scans use a top-down approach that starts with the entire genome, and may highlight genes that are fundamentally unknown. Single-cell sequencing has the potential to make developmentally relevant data accessible on a genome-wide level.
- *A change of focus from universals to specifics*. On a theoretical level, we ought to appreciate that a universal mechanism at the core of domestication might not exist, or might be limited in what traits and species it applies to. Accordingly, we ought to turn research towards particular traits, species, and their histories rather than chasing the universal. There have been many fruitful examples of research on domestic animals that focused in on specific traits that are relevant to different phases of domestication, *e.g.*, amylase activity in dogs (Axelsson *et al.* 2013) and introgressed resistance to gastrointestinal parasites in domestic goats (Zheng *et al.* 2020).

The neural crest cell hypothesis has had an influence beyond animal domestication, including on the literature on human evolution; see Sánchez-Villagra and van Schaik (2019) for a review of the history and current state of the self-domestication hypothesis. If there is a useful analogy between human evolution and domestication, it might be possible to learn about human evolution by studying the genetic basis of domestication. However, we would argue that any universal genetic mechanisms, neural crest-related or otherwise, proposed for domestication are currently not ready for export to other disciplines.

## Conclusions

In conclusion, the neural crest cell hypothesis is an explanation looking for a problem, and it is implausible in the light of modern quantitative genetics. The genomic evidence suggests that some variants near genes involved in the neural crest have been selected, but not that they were selected for their function in the neural crest, nor that they have pleiotropic effects that connect distinct domestication traits. Specifically, the genomic evidence highlights signaling pathways such as *Wnt* and *FGF* that contribute to many developmental processes besides the neural crest. Given the lack of knowledge about the functional genetics of early domestication, we suggest that it is premature to posit any unifying mechanism. 

## Conflicts of interest

None declared. 
